# Isolation of Huangpi tick virus 1 and assessment of potential zoonotic relevance

**DOI:** 10.1186/s13071-026-07282-8

**Published:** 2026-03-09

**Authors:** Liyan Fu, Chenxuan Li, Jun Ni, Jin Qian, Jian Xiao, Qiong Zhu, Shouwei Huang, Jinfeng Xiong, Qi Chen, Xuhua Guan, Rui Fang, Du Fen, Shuang Tang, Fei Deng, Dan Liu, Shu Shen, Xiaoli Wu

**Affiliations:** 1 Brain Science and Advanced Technology Institute, Wuhan University of Science and Technology, Wuhan, 430065 China; 2 State Key Laboratory of Virology and Biosafety and National Virus Resource Center, Wuhan Institute of Virology, Chinese Academy of Sciences, Wuhan, 430071 China; 3 Hubei Provincial Center for Disease Control and Prevention, Wuhan, China; 4 State Key Laboratory of Agricultural Microbiology, College of Veterinary Medicine, Huazhong Agricultural University, Hubei Province, Wuhan, 430070 People’s Republic of China; 5 Hubei Center for Animal Disease Control and Prevention, Hubei Province, Wuhan, 430070 People’s Republic of China

**Keywords:** *Orthonairovirus*, Huangpi tick virus 1, *Haemaphysalis longicornis*, Virus isolation, Zoonotic potential

## Abstract

**Background:**

The global burden of tick-borne viral diseases (TBVDs) has significantly increased in recent decades, emerging as a critical public health priority due to their diverse pathogenic profiles, severe disease outcomes, and therapeutic challenges. Within the expanding landscape of tick-borne pathogens, the *Orthonairovirus* genus has gained particular scientific attention for its members’ zoonotic capacity and clinical virulence*. Orthonairoviruses* are arboviruses that infect humans and animals, posing a serious threat for the spread of zoonotic diseases. Huangpi tick virus 1 (HpTV-1), a member of the genus *Orthonairovirus*, has been detected in ticks.

**Methods:**

*Haemaphysalis longicornis* collected in 2023 from Central China were pooled for RNA-seq and reverse transcription quantitative polymerase chain reaction (RT-qPCR) screening. Genomes were assembled and viruses isolated in suckling mice and Vero E6. Replication kinetics, mouse pathogenesis, and 223 livestock sera were assessed.

**Results:**

In the present study, we isolated HpTV-1 from *Haemaphysalis longicornis* in Central China. Viral genome and phylogenetic analyses placed HpTV-1 in a distinct clade close to the Songling and Tamdy viruses within the *Nairoviridae* family. In vitro experiments demonstrated that HpTV-1 infects a wide range of animal and human cell lines. Importantly, all infected C57BL/6 mice survived without overt signs of severe disease while developing only minor pathological lesions in specific organs (liver, spleen, and lungs). Serological surveys revealed HpTV-1 antibodies, including neutralizing antibodies, in 16.1% of the goats, suggesting that HpTV-1 can infect livestock.

**Conclusions:**

These findings suggest that HpTV-1 is an *Orthonairovirus* capable of infecting animals, highlighting its potential risk and the need for enhanced surveillance and research, particularly in Central China and other endemic regions.

**Graphical Abstract:**

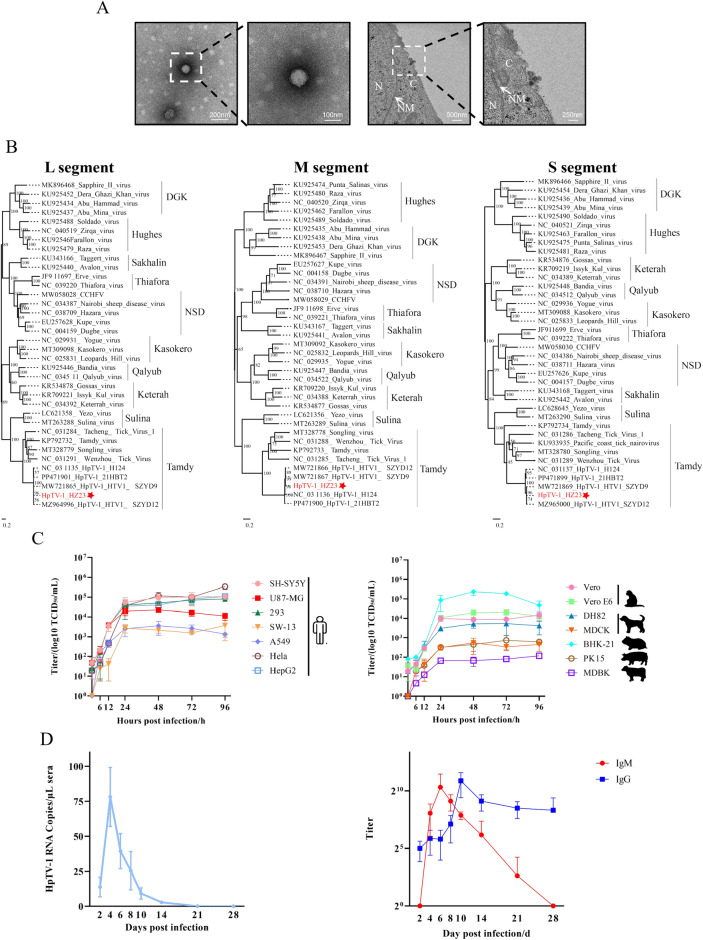

## Background

The global burden of tick-borne viral diseases has significantly increased in recent decades [1]. Among tick-borne pathogens, the *Orthonairovirus* genus has attracted significant attention for its zoonotic capacity and high fatality rate, including Crimean-Congo hemorrhagic fever virus (CCHFV), with case fatality rates of 10–40%, and Nairobi sheep disease virus (NSDV), which causes severe hemorrhagic gastroenteritis in ruminants [2–4]. The advent of molecular diagnostics has accelerated the identification of novel viruses within this genus, including Yezo virus (YEZV), Songling virus (SGLV), Beiji nairovirus (BJNV), Tacheng tick virus 1 (TcTV-1) and Wetland virus (WELV), all of which are associated with human febrile illness in Japan and China [5–10].

Huangpi tick virus 1 (HpTV-1), a recently characterized *Orthonairovirus*, was initially detected via metagenomic sequencing in ticks [11, 12]. Phylogenetic analysis revealed that HpTV-1 exhibits the closest evolutionary relationship with Tacheng tick virus 1 (TcTV-1), suggesting a similar zoonotic potential. However, the lack of isolated viral strains has substantially impeded characterization of its biological properties, host–pathogen interactions, and cross-species transmission potential. Given the propensity of tick-borne viruses for geographical expansion through host migration and ecological perturbations, comprehensive evaluation of HpTV-1’s infectivity and pathogenicity become imperative for public health risk assessment.

In this study, we successfully isolated an infectious HpTV-1 strain from *Haemaphysalis longicornis* (*H. longicornis*) in Central China and characterized its cellular tropism, pathogenic profile, and seroprevalence in animal reservoirs. Our study demonstrated that HpTV-1 poses a potential public health threat, warranting further attention.

## Methods

### Tick sample collection, viral genome sequencing

In July 2023, adult *H. longicornis* were collected from the wild by flagging vegetation and directly from asymptomatic animal hosts in the Dabie Mountain region. The ticks were divided into groups on the basis of collection site and engorgement status of the ticks, with each group comprising about 50 ticks. A total of 50 tick sample pools were randomly selected for library preparation. Total RNA was purified from the supernatant using the RNAiso Plus (Takara, Shiga, Japan). The purified RNA was used for library preparation using the VAHTS Universal V8 RNA-seq Library Prep Kit (Vazyme, Nanjing, China). Paired-end (2 × 150 bp) sequencing of each RNA library was performed using an Illumina NovaSeq 6000 System. For prevalence estimation, sample pools not subjected to high-throughput analysis were screened by reverse transcription quantitative polymerase chain reaction (RT-qPCR), with positive specimens subsequently characterized through nested PCR amplification and Sanger sequencing. Sequencing data quality control and viral sequence identification/assembly were conducted using standardized bioinformatics pipelines as previously described[13].

### Virus isolation and electron microscopy

Virus isolation was achieved through intracranial inoculation of suckling mice. A mixed homogenate of RNA-positive ticks was prepared and inoculated into 2-day-old Kunming (KM) suckling mice. Brain tissues were harvested and added to Modified Eagle’s medium (MEM, Gibco) containing 2% fetal bovine serum (FBS, Gibco), as previously described [14]. After homogenization using a tissue grinder (NewZongke, Wuhan, China), tissue fragments were removed by centrifugation at 8000 × g for 15 min at 4 °C. The supernatant was then diluted with MEM medium with 2% FBS in two gradients (v/v = 1:20 and 1:50) and inoculated onto Vero E6 cells. The cells were adsorbed for 2 h at 37 °C with 5% CO_2_, after which the medium was switched to MEM containing 2% FBS and cultured for 3–4 days at incubator. For subsequent passaging, cells from the first passage were diluted threefold, and this procedure was repeated for further passages. Viral infection in each cell generation was detected by immunofluorescence assay (IFA) using HpTV-1 NP polyclonal antibody (α-HpTV-1-NP), which was used as the primary antibody. The production of virus from infected cells and its release into the supernatant were assessed using reverse transcription quantitative polymerase chain reaction (RT-qPCR), and the primer sequences were recorded in Table S1. All RT-qPCR assays were performed using the One Step TB Green® PrimeScript™ PLUS RT-PCR Kit (Perfect Real Time) (Takara, Shiga, Japan).

Viral particles were concentrated and purified from the culture supernatant, and the fraction containing viral particles was collected and applied to grids for negative-staining electron microscopy (EM), as previously described [15]. Ultrathin sections were prepared by fixing HpTV-1-infected cells with 2.5% (w/v) glutaraldehyde in 0.1 M sodium phosphate solution. Transmission electron microscopy (TEM) was employed to observe the intracellular viral particles.

### Cell infection and one-step growth curve assays

In this study, the cell lines were procured from the American Type Culture Collection (ATCC) and were cultured by National Virus Resource Center (NVRC) (Table S2). The cells were infected with HpTV-1 at a multiplicity of infection (MOI) of 1 and maintained at 37 ℃ for 3 days. The viral infection of the cells was assessed using an IFA, utilizing α-HpTV-1-NP as the primary antibody.

To elucidate the one-step growth kinetics of the virus, 50 μL aliquots of supernatant were harvested from the infected cell cultures at specified time intervals. The viral titer in the supernatant was subsequently determined by an end-point dilution assay on Vero E6 cells.

### Serological, histopathological, and immunohistochemical analysis of infections in mice

Six adult C57BL/6 mice were inoculated intraperitoneally with 100 μL of viral supernatant containing 10^6^ TCID_50_ of HpTV-1, and the control group received 100 μL of MEM supplemented with 2% FBS. Serum samples were collected from mice on 2, 4, 6, 8, 10, 14, 21, and 28 days postinfection (dpi). The viral load in the serum was quantified by standard curve-based RT-qPCR. Vero E6 cells infected with HpTV-1 at 1 MOI for 72 h were used as antigen for IFA and enzyme-linked immunosorbent assay (ELISA) antibody detection. The titers of immunoglobulin (Ig)G and IgM antibodies were measured by ELISA, using 3,3′,5,5′-tetramethylbenzidine (TMB) chromogen solution and acid-free stop solution for TMB substrate (450nm) (Beyotime, Shanghai, China). The optical density (OD) value was measured using dual wavelength detection at 450/630 nm. At 14 dpi, serum samples from HpTV-1-infected mice were subjected to neutralization assays to assess their neutralizing activity against HpTV-1. Tissue samples were collected from mice with the highest viral load at 4 dpi, fixed in 4% paraformaldehyde for 24 h, and then embedded in paraffin. The tissues were sectioned continuously at 5-μm thickness and stained with hematoxylin-and-eosin (H&E). Immunohistochemical (IHC) staining was performed using α-HpTV-1-NP as the primary antibody to detect the distribution of viral antigens in the mouse tissues.

### Serological examination of animal serum samples

A total of 223 serum samples from healthy animals were collected in central China, with background information such as the collection sites detailed in Table 1. The antigen of the HpTV-1 NP protein was prepared using the luciferase immunoprecipitation system (LIPS) and co-incubated with the sera for detection, with the specific method described in a previous study [16]. The IFA was employed, using animal serum at a 1:20 dilution as the primary antibody and Fluorescein Isothiocyanate (FITC) Donkey Anti-Goat IgG (H + L) (Abclonal, Wuhan, China) or Rabbit Anti-Cow IgG H&L (FITC) (Abcam, Wuhan, China) as the secondary antibody to detect antibodies in the sera, with Hoechst 33,258 (Beyotime, Shanghai, China) used for nuclear staining. Among these, antibody-positive samples were diluted at ratios of 1:8, 1:16, and 1:32, respectively, and incubated with 100 TCID_50_ of HpTV-1 to detect neutralizing antibodies, with each sample repeated three times, following the procedure described in a previous study [17].

**Table 1:** Background information and test result for animal serum samples

Location	Species	Serum samples	Positive antibodies against HpTV-1 detected by LIPS (%)	Positive antibodies against HpTV-1 detected by IFA (%)	Neutralization tests	
					HpTV-1 (%)	End-point titer
A	Goat	53	20 (37.7)	19 (35.8)	0	
B	Goat	50	8 (16)	6 (12)	0	
C	Goat	30	10 (33.3)	10 (33.3)	3 (10)	2^3^
	Cattle	20	0	0	0	
D	Goat	30	2 (6.7)	1 (3.3)	2 (6.7)	2^4^
	Cattle	10	0	0	0	
	Pheasant	30	0	0	0	
Total		223	40 (17.9)	36 (16.1)	5 (2.2)	

### Bio-informatics analyses

Sequence editing and organization were conducted using Notepad++ software. The maximum likelihood (ML) phylogenetic tree was constructed with phyloSuite v1.2.3, validated through 1000 bootstrap replicates, and refined using FigTree v1.4.4. The antibody test results were analyzed by the GraphPad Prism software version 8.0.2 (San Diego, CA, USA).

## Results

### Isolation and identification of an *Orthonairovirus* from *H. longicornis* in Central China

**Figure 1:**
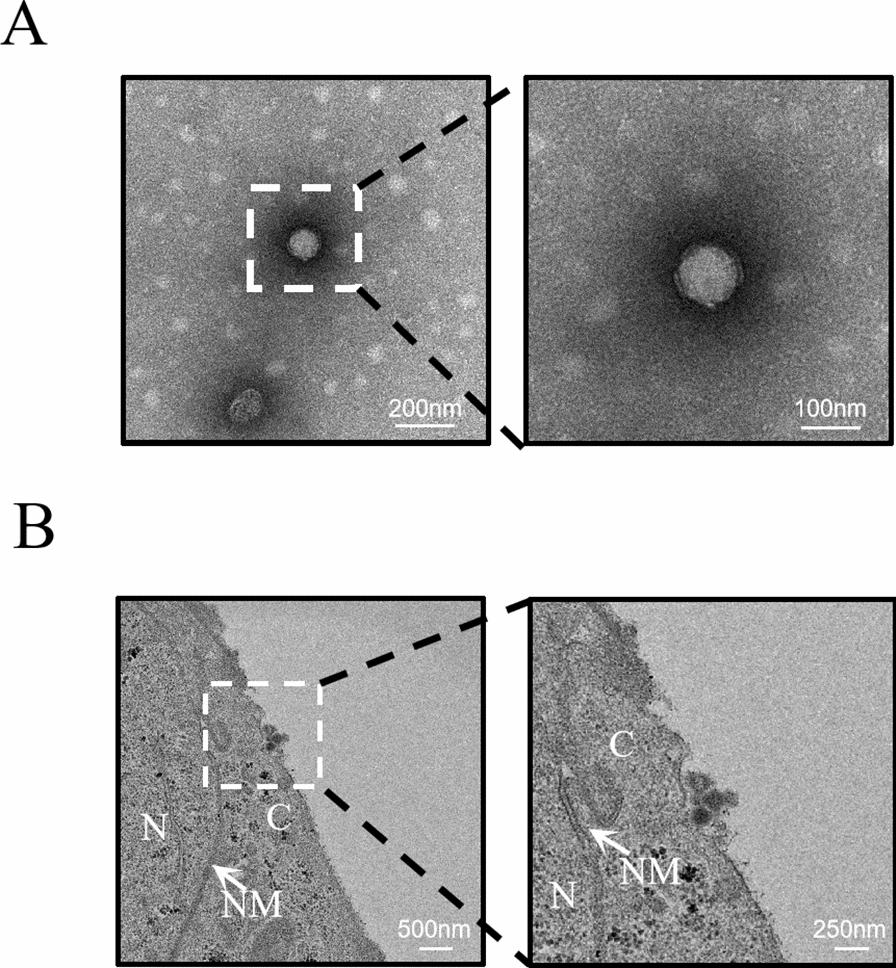
Isolation and morphological characterization of HpTV-1. **A** Negative-staining electron microscopy (EM) analysis of purified virions from culture supernatant. **B** The image obtained by transmission electron microscopy (TEM) shows that virus particles were located in cytoplasm of infected Vero E6 cells. N, nucleus; C, cytoplasm; and NM, nuclear membrane

To investigate the virome composition of ticks in Central China, we conducted high-throughput sequencing of pooled tick samples. From one of these pools, we obtained three reads related to HpTV-1 assembled into three contigs (large (L), middle (M), and small (S) segments). The ticks were identified as *H. longicornis* on the basis of partial sequences of the mitochondrial 16S rRNA gene (Fig. S1). HpTV-1 HZ23 strain was successfully isolated from the homogenate of the positive sample pool, and green fluorescence (confirming the expression of HpTV-1 nucleoprotein (NP)) was observed in infected cells across multiple passages (P1–P4) (Fig. S2). The cycle threshold (CT) values of viral RNA in the supernatants and cells at different passages were detected using RT-qPCR, indicating viral amplification (Fig. S3). Spherical, enveloped virus particles approximately 80–120 nm in diameter were observed using negative-staining electron microscopy (Fig. 1A). Budding virus particles were observed in the cytoplasm of infected cells (Fig. 1B).

### Molecular identification and phylogenetic analysis revealed that HpTV-1 belongs to the Tamdy group

**Figure 2:**
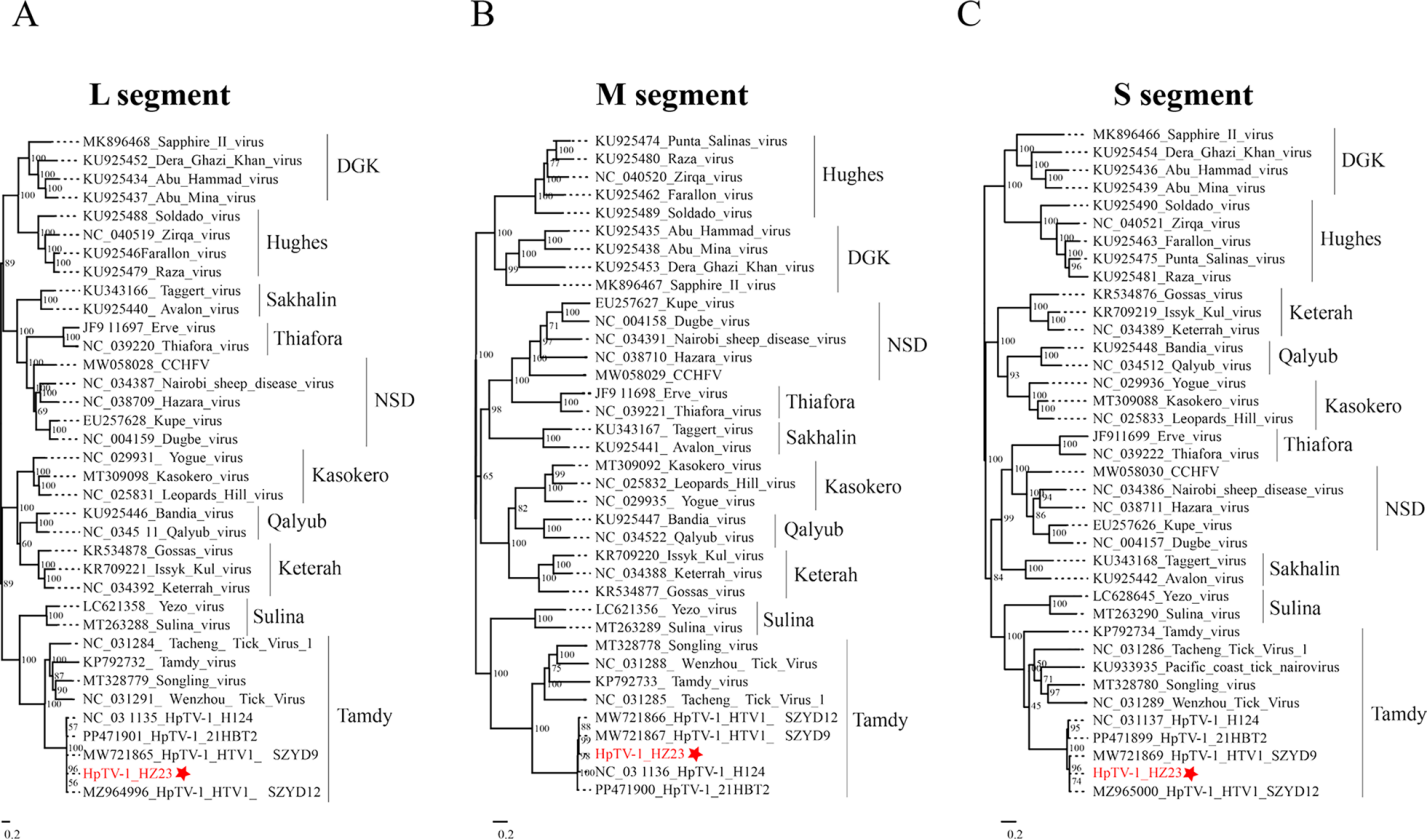
Phylogenetic placement of HpTV-1 within the Tamdy group. Maximum-likelihood trees were inferred from nucleotide sequences of the L (**A**), M (**B**), and S (**C**) segments. Numbers at nodes denote bootstrap support (1 000 replicates); scale bars indicate substitutions per site; branch lengths are proportional to genetic distance. Confirmation that HpTV-1 HZ23 strain belongs to the Tamdy group

The genome sequence of the HpTV-1 HZ23 strain was determined, comprising three segments: L (11,948 nucleotides, encoding RNA-dependent RNA polymerase), M (4771 nucleotides, encoding glycoprotein), and S (1924 nucleotides, encoding NP), which were deposited at the Science Data Bank (ScienceDB) under accession number: 10.57760/sciencedb.30690. Phylogenetic analysis demonstrated that HpTV-1 is a member of the genus *Orthonairovirus* within the family *Nairoviridae* and is most closely related to SGLV, TAMV, TcTV-1, and Wenzhou tick virus (WzTV) (Fig. 2A–C), forming a distinct branch within the Tamdy group [18]. Notably, HpTV-1 HZ23 strain showed the highest sequence similarity with SZYD9 in the L segment (99.8%), with SZYS9 and SZYD12 in the M segment (99.7%), and with SZYD12 in the S segment (99.7%) (Table S3).

### A variety of cells from both animals and humans are susceptible to infection and replication by HpTV-1

**Figure 3:**
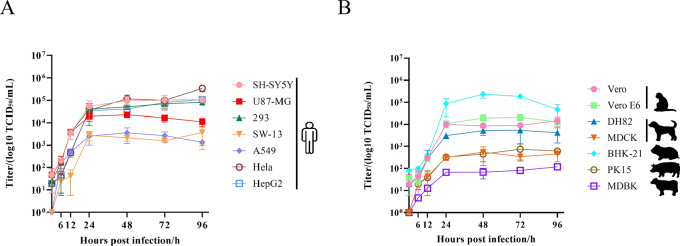
One-step growth curves of HpTV-1 in different cell lines. **A** Human cells and **B** animal cells were infected with HpTV-1 at one multiplicity of infection (MOI) and supernatants were harvested at the indicated time points. The viral titers in supernatants were determined as described

HpTV-1 showed a broad cellular tropism (Fig. S4). It successfully infected seven human-derived cell lines: SH-SY5Y, U87-MG, 293, SW-13, A549, HeLa, and HepG2, as well as cell lines from monkey (Vero, Vero E6), dog (DH82, MDCK), hamster (BHK-21), pig (PK15), and cow (MDBK) (Fig. S4). One-step growth curve analyses indicated that viral titers reached a steady-state plateau at 24 h postinfection (hpi) in different cell lines. HpTV-1 replicated efficiently in BHK-21, HeLa, SH-SY5Y, HepG2, and 293 cells, reaching titers of approximately 10^5^ TCID_50_/mL (Fig. 3); however, it performed poorly in PK15, MDCK, and MDBK cells, where titers remained below 10^3^ TCID_50_/mL. Considering the demonstrated infectivity of HpTV-1 in various cell lines, its zoonotic potential requires careful monitoring.

### HpTV-1 induces pathological lesions in adult C57BL/6 mice

**Figure 4:**
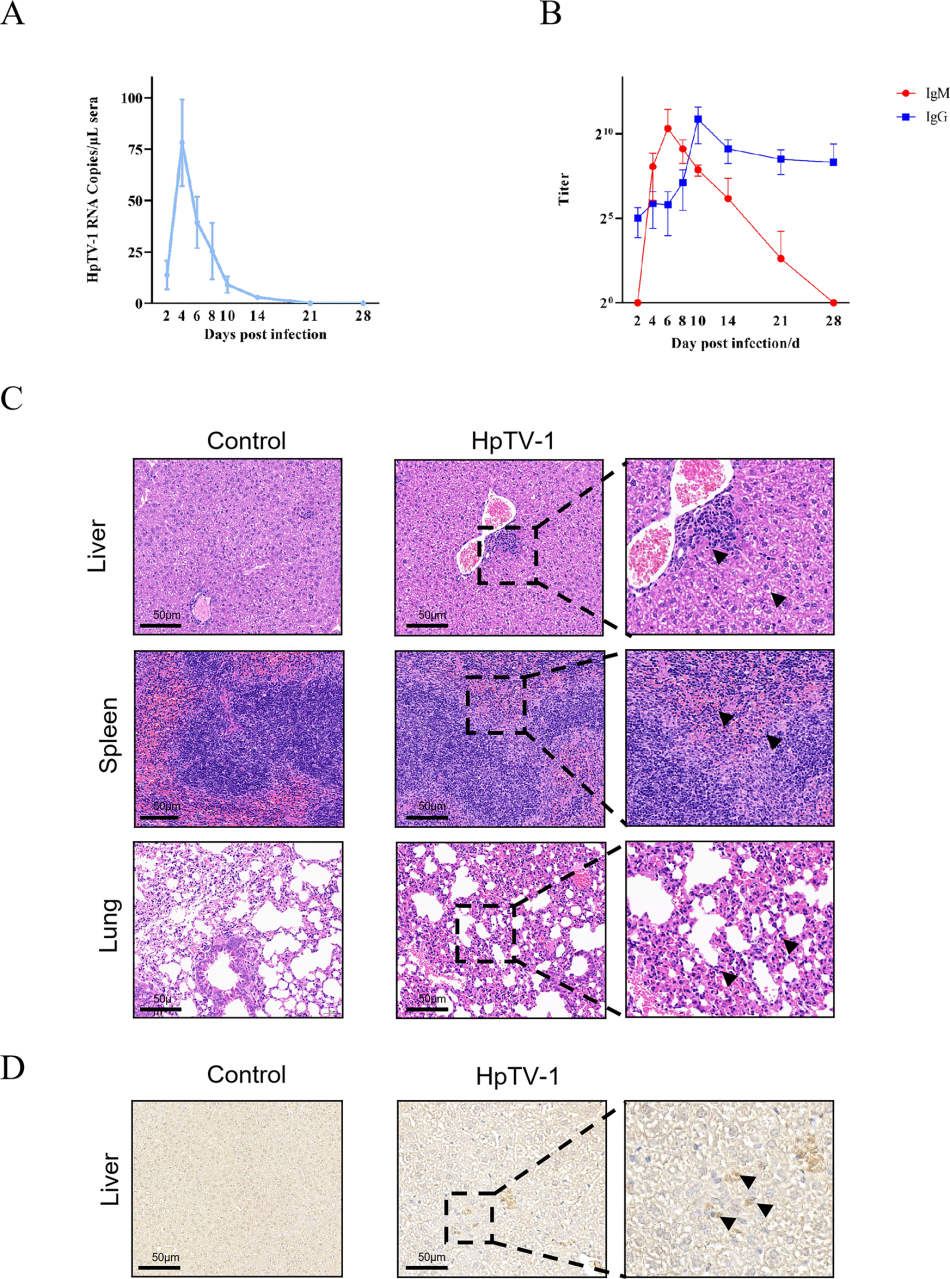
HpTV-1 infection dynamics and pathology in adult C57BL/6 mice. **A** RT-qPCR detection of HpTV-1 RNA copies in sera collected on the indicated day after challenge. Data are presented as the mean ± SD, *n* = 6. **B** IgM and IgG antibody expression in the sera of mice collected on the day following challenge. Sera were serially diluted twofold using an endpoint dilution assay. Titers were reported as the reciprocal of the highest dilution whose OD was at least threefold greater than that of the negative control. The antibody titers are shown with the corresponding symbols with means ± SD. **C** Representative H&E-stained sections at 6 dpi showing focal hepatic necrosis (arrowheads) and mild splenic/alveolar granulocyte infiltrates (arrows). Scale bars, 50 µm. **D** Immunohistochemistry demonstrating HpTV-1 nucleoprotein (brown) in hepatocytes. Negative-control sections lacked staining. Scale bar, 100 µm

No lethality or significant clinical symptoms were observed in C57BL/6 mice (6–8 weeks old) following intraperitoneal inoculation with HpTV-1 (10^6^ TCID_50_/mouse) during the 28-day observation period, mirroring the nonlethal profile of CCHFV in the same model [19], and suggesting a similarity in their pathogenic mechanisms. HpTV-1 infection caused transient viremia, exhibiting a typical kinetic pattern with a peak on day 4 that subsequently resolved and became undetectable by day 14 (Fig. 4A). All mice stimulated a humoral immune response, with IgM antibodies detected on day 4 and peaking on day 6, whereas IgG antibodies were expressed at low levels on day 2, peaking on day 10, and stabilizing after day 14 (Fig. 4B). Serum samples collected at 14 days postinfection (dpi) demonstrated detectable neutralizing antibody titers with an efficacy exceeding 60% at a 2^–5^ dilution (Fig. S5), which potentially contributed to viremia resolution. These findings confirmed that HpTV-1 is capable of inducing a robust humoral immune response, indicating active viral replication and systemic infection in vivo. Histopathological observations revealed focal necrosis of hepatocytes with nuclear coalescence and fragmentation along with lymphocyte infiltration into the liver lobules at 6 dpi (Fig. 4C). Granulocyte infiltration was observed in the spleen and alveolar walls, and immunohistochemical analysis revealed HpTV-1 NP antigen expression in the mouse liver (Fig. 4D). Notably, viral antigen expression was detected in the hepatic tissues, suggesting that the liver may serve as a primary target organ for HpTV-1 infection. This antigen may trigger localized or systemic immune responses, potentially influencing disease progression. These results highlighted the potential pathogenicity of HpTV-1 and its possible implications for animal health.

### Surveys of the prevalence of HpTV-1 in *H. longicornis* and animal sera suggest a potential risk of infection

Epidemiological surveillance identified HpTV-1 RNA in 10 pools of 145 *H. longicornis* groups from the Central China, and all positive samples were amplified using RT-PCR (Fig. S6). The detection of HpTV-1 in ticks [11, 12] suggests its presence in these regions, warranting increased attention. Serological analysis of 223 animal (goat, cattle, and pheasant) serum samples with tick bite histories revealed IgG antibodies against HpTV-1 in 36 goat serum samples (positivity rate: 16.1%) from the four cities (Table S4 and Fig. S7). Neutralization assays demonstrated that five goat serum samples exhibited discernible neutralizing activity, with a neutralizing activity endpoint titer of 2^3^–2^4^ (Table 1). The detection of HpTV-1 neutralizing antibodies in goats confirmed the circulation of the virus in potential reservoir hosts, indicating a likely underestimation of its prevalence and highlighting the need for enhanced surveillance of infection risks.

## Discussion

In recent years, emerging tick-borne pathogens have posed an increasingly severe threat to human health. As novel viruses continue to emerge, research on their potential pathogenic risks can lay a crucial foundation for the prevention and control of emerging tick-borne infectious diseases. We report the first isolation of an *Orthonairovirus*, HpTV-1, from *H. longicornis* in Central China. Phylogenetic analyses based on L, M, and S genome sequences further positioned HpTV-1 within the Tamdy virus group (Fig. 2), clustering closely with other recently identified *Orthonairovirus* such as SGLV, TAMV, WzTV, and TcTV-1. The high degree of nucleotide identity between the newly isolated HpTV-1 strain and previously reported sequences from tick metagenomic studies (Table S3) further supports its genetic stability and possible regional circulation.

While TAMV replication in Vero cells resulted in notable cytopathic effects[20], SGLV demonstrated a broader cell tropism, supporting replication in SMMC-7721, BHK-21, and Vero cell lines [19]. Our findings demonstrate that HpTV-1 can infect a broad spectrum of cell lines derived from human, nonhuman primate, canine, rodent, porcine, and bovine species. However, its replication efficiency exhibited significant host dependence, with peak viral titers reaching 1.16 × 10^5^ TCID_50_/mL in permissive cell lines such as BHK-21 and HeLa, while being markedly reduced to 5.42 × 10^2^ TCID_50_/mL in PK15 and MDBK cells (Fig. 3). Given the demonstrated infectivity of HpTV-1 in human cell lines, its zoonotic potential warrants careful monitoring.

In previously established mouse models for viruses of the genus *Orthonairovirus*, C57BL/6J mice infected with CCHFV developed hemolysis and aplastic anemia by day 3 but survived [19]. While immunocompetent BALB/c mice were asymptomatic following TAMV infection, IFNAR^−/−^ mice succumbed to high-dose viral challenge[22]. Similarly, type I/II interferon receptor double-knockout (AG129) mice infected with YEZV died from fulminant hepatitis with predominant liver and spleen viral replication, but BALB/c and C57BL/6 were monitored for 14 days after the viral challenge [23]. Thus, the majority of *Orthonairovirus* do not cause lethal infection in C57BL/6 and other immunodeficient mice but replicates primarily in the liver and spleen. This is consistent with our finding that HpTV-1 infection was nonlethal in this model (Fig. 4). Viral RNA copies have been detected in the sera of mice infected with CCHFV, TAMV, and YEZV[19, 22, 23], which is consistent with our findings that HpTV-1 infection can also result in detectable viremia (Fig. 4A). Similar to TAMV infection in mice [22], HpTV-1 infection elicited the production of both IgM and IgG antibodies (Fig. 4B), which exhibited neutralizing activity against HpTV-1, and may contribute to the clearance of viremia. Notably, IgG antibodies against HpTV-1 were detected in 16.1% of animal serum samples, with a subset of these samples exhibiting neutralizing activity (Table 1), indicating previous exposure and the potential for protective immune responses in this livestock population. These findings suggest that the current prevalence of HpTV-1 in Central China may be underestimated, warranting heightened surveillance due to its potential infection risks.

Despite the significant findings of this study, several limitations must be addressed. The lack of clinical data on seropositive individuals hinders the assessment of disease associations. The exclusive use of a mouse model and absence of data on viral shedding or transmission dynamics restrict our ecological understanding. Future studies should prioritize the study of HpTV-1, including long-term surveillance of *H. longicornis* and domestic animals, and clarify transmission routes and pathogenicity in humans and livestock.

## Conclusions

In summary, we first isolated an *Orthonairovirus*, HpTV-1, from *H. longicornis* in Central China. This virus exhibits broad cross-species infectivity in vitro and establishes non-lethal infection with viraemia and neutralizing antibody responses in mice. Serological surveys further demonstrate its active circulation among local animal populations, confirming exposure in livestock. These findings underscore its zoonotic potential and warrant enhanced surveillance to assess public health risks in endemic regions

## Data Availability

Data supporting the main conclusions of this study are included in the manuscript.

## Abbreviation

TBVDs: Tick-borne viral diseases
HpTV-1: Huangpi tick virus 1
CCHFV: Congo hemorrhagic fever virus
NSDV: Nairobi sheep disease virus
YEZV: Yezo virus
SGLV: Songling virus
BJNV: Beiji nairovirus
TcTV-1: Tacheng tick virus 1
WELV: Wetland virus
*H. longicornis*: *Haemaphysalis longicornis*
KM: Kunming
MEM: Modified Eagle’s medium
FBS: Fetal bovine serum
IFA: Immunofluorescence assay
RT-qPCR: Reverse transcription quantitative polymerase chain reaction
EM: Electron microscopy
TEM: Transmission electron microscopy
ATCC: American Type Culture Collection
NVRC: National Virus Resource Center
MOI: Multiplicity of infection
dpi: Days postinfection
ELISA: Enzyme-linked immunosorbent assay
OD: Optical density
H&E: Hematoxylin and eosin
IHC: Immunohistochemical
LIPS: Luciferase immunoprecipitation system
ML: Maximum likelihood
L: Large
M: Middle
S: Small
NP: Nucleoprotein
CT: Cycle threshold
WzTV: Wenzhou tick virus
